# Bioresponsive Nanomaterials: Recent Advances in Cancer Multimodal Imaging and Imaging-Guided Therapy

**DOI:** 10.3389/fchem.2022.881812

**Published:** 2022-03-18

**Authors:** Zeng Zeng, Huali Gao, CongXian Chen, Lianbo Xiao, Kun Zhang

**Affiliations:** ^1^ Orthopedic Surgery Department, Institute of Arthritis Research in Integrative Medicine, Shanghai Academy of Traditional Chinese Medicine, Guanghua Hospital Affiliated to Shanghai University of Traditional Chinese Medicine, Shanghai, China; ^2^ Cancer Center, Department of Ultrasound Medicine, Zhejiang Provincial People’s Hospital, Affiliated People’s Hospital, Hangzhou Medical College, Hangzhou, China; ^3^ Central Laboratory, Shanghai Tenth People’s Hospital, Tongji University School of Medicine, Shanghai, China

**Keywords:** nanomaterials, cancer, imaging, photothemal, photodynamic therapy

## Abstract

Cancer is a serious health problem which increasingly causes morbidity and mortality worldwide. It causes abnormal and uncontrolled cell division. Traditional cancer treatments include surgery, chemotherapy, radiotherapy and so on. These traditional therapies suffer from high toxicity and arouse safety concern in normal area and have difficulty in accurately targeting tumour. Recently, a variety of nanomaterials could be used for cancer diagnosis and therapy. Nanomaterials have several advantages, *e.g.*, high concentration in tumour via targeting design, reduced toxicity in normal area and controlled drug release after various rational designs. They can combine with many types of biomaterials in order to improve biocompatibility. In this review, we outlined the latest research on the use of bioresponsive nanomaterials for various cancer imaging modalities (magnetic resonance imaging, positron emission tomography and phototacoustic imaging) and imaging-guided therapy means (chemotherapy, radiotherapy, photothermal therapy and photodynamic therapy), followed by discussing the challenges and future perspectives of this bioresponsive nanomaterials in biomedicine.

## Introduction

Cancer is one of malignant diseases that can happen in different parts of the body (Barreto et al.,2011). It causes abnormal cells division and various malignant behaviors such as cancer cells uncontrolled growth, invasion, metastasis and immortality (Barreto et al.,2011). Gene mutations that control cell cycles are mostly associated with cancer progression (Husband & Reznek, 2000). There are a variety of factors can trigger gene mutations such as exposure to ultraviolet light, infectious pathogens and so on (Rai et al.,2021). Cancer is an extremely complex disease and its treatment is still unspecific. Chemotherapy, radiotherapy, immunotherapy and surgery are main treatment methods for cancer (Mahasneh et al.,2017). Drug resistance is a major obstacle in cancer treatment due to epigenetic changes, upregulated drug efflux and many cellular mechanisms (Holohan et al.,2013; Turner & Reis-Filho, 2012). Both chemotherapy and radiotherapy have side effects on healthy tissues and poor specificities for cancer tissues. For instance, the common chemotherapy agents such as doxorubicin and paclitaxel can exhibit anti-cancer effects and kill normal cells as well (Yoo et al.,2000)I, which is inevitable to cause many side effects due to its non-selective cytotoxic effects. Every year, cancer-arised deaths account for about 13% of all deaths, cancer-related mortality is expected to rise to about 13.1 million until 2030 (Tan et al.,2020). Therefore, cancer has become a huge health burden wordwide (Vogelstein et al.,2013).

Nanomaterials (NMs)-based cancer diagnostics is a relatively-new field, which can be used to diagnose cancer rapidly, regenerate drug delivery tissue, develop new medical products and enhance the efficiency of cancer treatment (Chen et al.,2016; Shapira et al.,2011). Currently, nanomaterials can be divided into many types according to different internal or external stimuli such as mechanical, magnetic, electrical, optical and biological ones (Wei et al.,2019; Zhang et al.,2019). Nanomaterials have many advantages such as nanoscale size, high surface-to-volume ratio, promising and controlled drug release profiles, and the robust ability to differentiate and eradicate malignant cells selectively (Tran et al.,2017; Wang et al.,2019). Because of these advantages, nanotechnology has attracted interests in cancer therapeutics. For instance, many nanovehicle platforms (10–200 nm) are favorable for loading drugs, specifically targeting tumor tissues and entering tumor cells (Davis et al.,2008; Duncan, 2003). This review aims to discuss different types of nanomaterials used in different imaging modalities with an highlight on the applications of bioresponsive nanomaterials in cancer imaging-guided therapy.

## Brief Overview of Different Kinds of Nanomaterials

**FIGURE 1:**
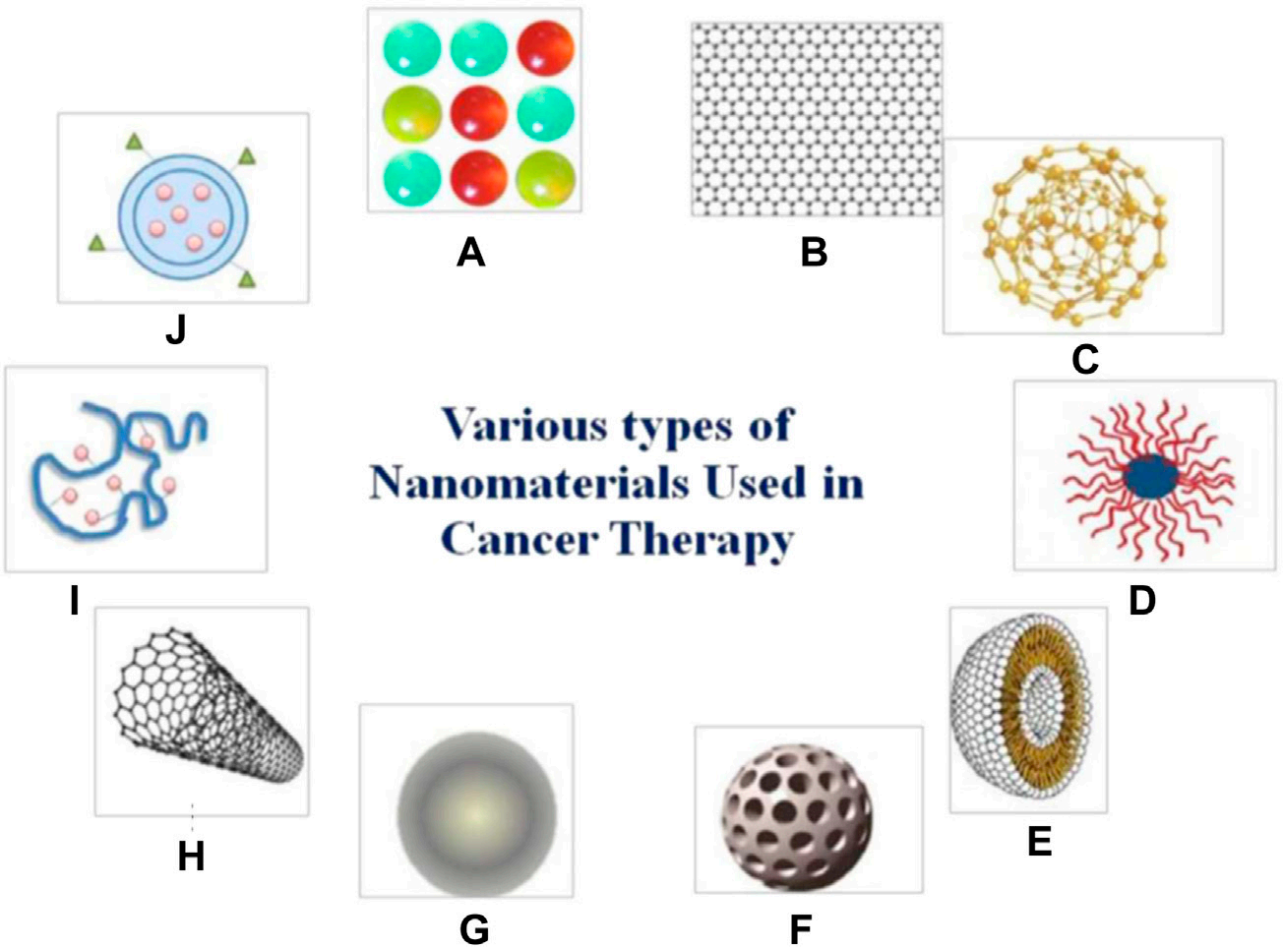
Different types of nanomaterials which are used in cancer treatment **(A)** quantum dots. **(B)** grapheme. **(C)** gold nanoparticles. **(D)** polymeric micelles. **(E)** liposomes. **(F)** silica nanoparticles. **(G)** magnetic nanoparticles. **(H)** carbon nanotubes. **(I)** polymer-drug conjugates. **(J)** polymeric nanoparticles. Reprinted (adapted) with permission from Rai et al. (2021). Medicina (Kaunas). 2021,57 (2),91. Copyright 2021 by the authors.

Nanomaterials are chemical substances including Nanoparticles, nanosphere, nanostars, nanorods and nanoshells (Siddique & Chow, 2020). Nanomaterials are used in many fields, *e.g.*, drug carriers, chemotherapy agents, photoacoustic agents, molecular manufacturing, photothermal agents, radiation dose enhancers, materials reactivity, biomarker discovery substances, molecular target therapy, biomedical imaging and so on (Hull et al.,2014; Mehta et al.,2019). Various types of nanomaterials can be used in cancer treatment such as quantum dots, graphene, gold nanoparticles, polymeric micelles, liposomes, silica nanoparticles, magnetic nanoparticles, carbon nanotubes, polymer-drug conjugates, and polymeric nanoparticles (Rai et al.,2021) (Figure 1).

In recent years, nanotechnology made tremendous progress in developing many nanostructured materials for diagnosis and treatment of cancer (Couvreur & Vauthier, 2006). Nanomaterials have a variety of unique advantages compared with other conventional anticancer agents. First, nanomaterials feature small size, approximately 100–1,000 times smaller than the size of a cancer cell. As a result, nanomaterials exhibit higher intracellular uptake and favorable targeting drug delivery (Goldberg et al.,2007). Second, nanomaterials hold great potential to overcome many limitations of the conventional chemotherapeutic agents. As a paradigm, paclitaxel is one of chemotherapeutic agents, which can treat breast, ovarian and other cancers (Lee et al.,2009). However, it has poor solubility and cannot be dissolved in aqueous solution readily (Bae et al.,2007). Nanovehicles such as liposomes and polymeric micelles impart them with an ability to enhance water solubility of theses hydrophobic drugs by incorporating them in the hydrophobic microenvironments (Hubbell, 2003). Third, owing to the enormous surface area, nanomaterials can carry many imaging and therapeutic agents. It is reported that a polymeric nanoparticle with an average diameter of 70 nm has the ability to accommodate almost 2000 drug molecules (Bartlett & Davis, 2007). Such a high drug loading capacity significantly improves the therapeutic efficacy of chemotherapeutic agents against cancer.

Additionally, nanomaterials paly a vital role in medical imaging such as magnetic resonance imaging (MRI), positron emission tomography (PET) and phototacoustic imaging. It is of great significance using iron oxide nanoparticles in T1-weighted and/or T2-weighted MRI and radioisotope chelator-free particles in PET (Rosado-de-Castro et al.,2018). Over the past few years, although nanomaterials have made some success in imaging and treatment of cancer to some extent, there are still many obstacles in its clinical application. For example, MRI has a high-resolution but its sensitivity is low; radioisotope imaging has high sensitivity but a relatively poor resolution is inevitable. Therefore, single imaging modality cannot offer comprehensive data, multiple imaging combining is expected to enhance cancer imaging systems (Burke et al.,2017).

Nanomaterials offer lots of opportunities to many cancer therapies such as chemotherapy, radiotherapy, photothermal therapy (PTT), photodynamic therapy (PDT), chemodynamic therapy and starving therapy due to their high permeability and retention. Because of the high surface area, nanomaterials can load or co-load a large number of different drugs. Thus nanomaterials are helpful as therapeutic carriers for realizing synergistic therapy such as PTT, PDT. Compared to monotherapy, nanomaterials avoid drug resistance effectively. Scientists made great efforts to combine cancer diagnosis with treatment using nanomaterials (Her et al.,2017).

## Applications of Bioresponsive Nanomaterials in Multimodal Imaging Diagnosis

### Magnetic Resonance Imaging

MRI is used as a non-invasive imaging method that is especially appropriate for soft tissue based on the property of nuclear magnetic resonance (Harisinghani et al.,2003). After excitation by a selective radio-frequency pulse, the active nuclei will be relaxed rapidly and then return to their initial state in MRI procedure. This relaxation procedure is divided into two parts, *i.e.*, longitudinal relaxation (T1) and transverse relaxation (T2). Both can generate magnetic resonance images for discriminating different tissues. Although MRI is a relatively expensive and time-consuming tool, it has enormous advantages such as high spatial resolution in three-dimensions, high contrast of soft tissues, facile extractions of physiological, anatomical and molecular information (Veiseh et al.,2010). Gadolinium (Gd^3+^) chelates that behave as paramagnetic complexes are usually used in MRI contrast agents, *e.g.*, Gd-DTPA is widely used in recent years (Tamada et al.,2009). However, repeated injections are needed with high chelates dosage to elongate blood circulation time, which, nevertheless, inevitably brought about some inaccuracies due to the false positive contrast enhancement (Huang et al.,2012).

**FIGURE 2:**
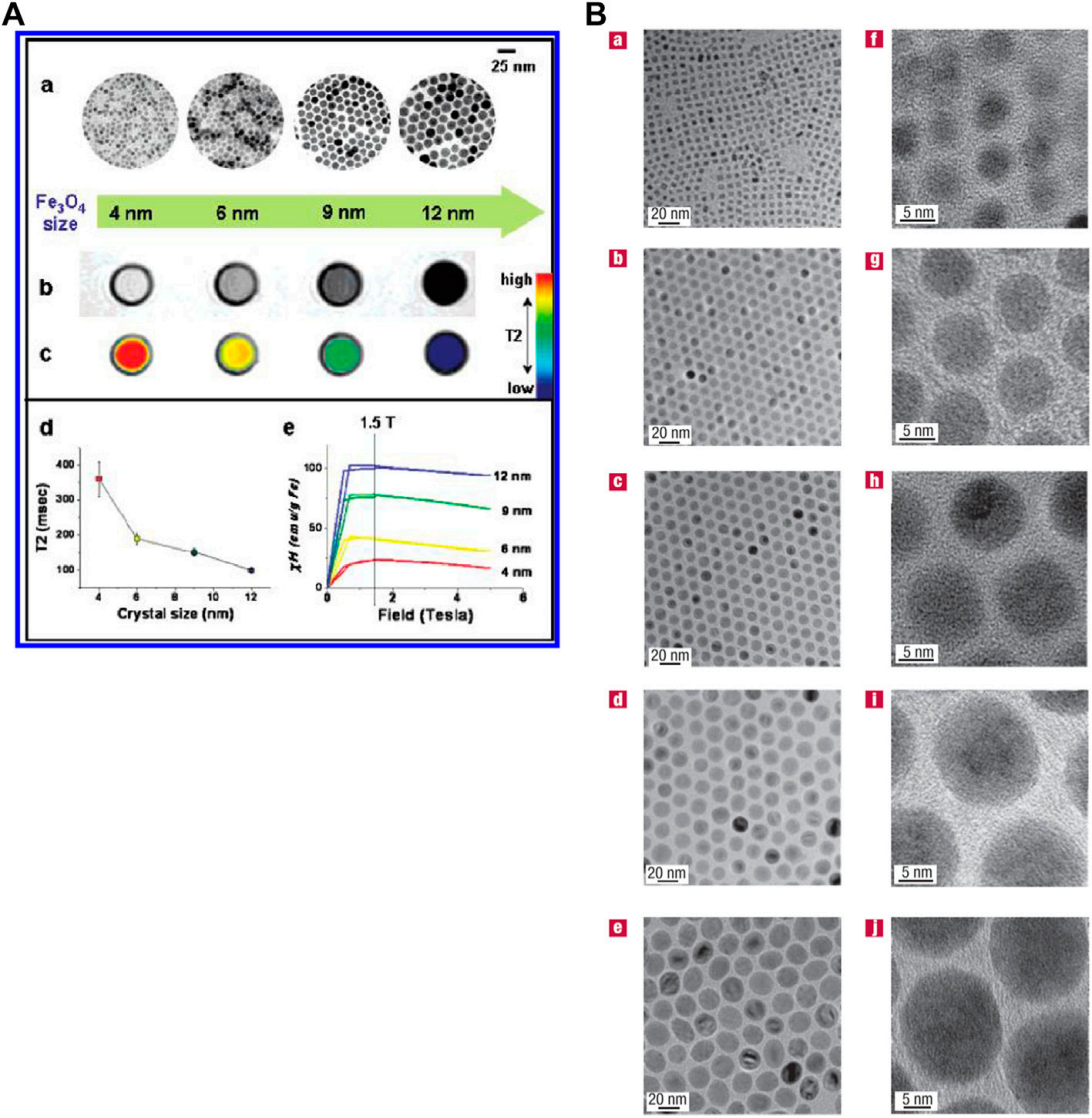
**(A)** Water-soluble Fe_3_O_4_ nanocrystals’ size effects on magnetism and MR signals. **(B)** Transmission electron microscope images of different oleic-Fe_3_O_4_ nanoparticles. Reprinted (adapted) with permission from Park et al. (2004). Nat Mater. 2004,3 (12),891-5. Copyright 2004 Nature Publishing Group. Reprinted (adapted) with permission from Jun et al. (2005). J Am Chem Soc. 2005,27,127 (16),5732-3. Copyright 2005 American Chemical Society.

MRI contrast agents that refer to nanomaterials with uniform size, enhanced relaxation properties and biocompatibility attract scientists’ eyes (Jun et al., 2005; Park et al.,2004). Food and Drug Administration (FDA) approved super paramagnetic iron oxide (SPIO) as a nanoparticle contrast agent in clinical trials (Thorek et al.,2006). SPIO is now widely used in T_2_ contrast agents. Many nanoparticles such as NiFe_2_O_4_, MnFeO_4_ and CoFe_2_O_4_ have also been proved for T_2_ contrast agents (Khemtong et al.,2011). Nanoparticle Gd-based contrast agents such as Gd_2_O_3_, GdPO_4_ and GdF_3_ can enhance the signal of T1-weighted MRI (Figure 2) (Jun et al., 2005; Jung et al., 2009; Park et al., 2004; Yin et al., 2012; Zhang et al., 2011). Intriguingly, ultrasmall-sized iron oxide nanoparticles (ESIONs) with sizes less than 4 nm have been proved as good candidates for T_1_-weighted imaging (Kim et al.,2011). However, these novel nanoparticles are still in the stage of preliminary animal studies *in vivo* for MRI. There is a long way to before entering human before addressing their biocompatibility and pharmacokinetics concerns (Huang et al.,2012).

### Positron Emission Tomography

PET is a nuclear medicine imaging method, and it can provide quantitative and sensitive readout of an administered radiotracer in order to evaluate the targeting pharmacokinetics and efficiency objective to tissues or organs (Ametamey et al.,2008; Shokeen & Anderson, 2009). Gold nanoparticles are mostly used nanomaterials in PET. Radioiodine-124-labelled tannic acid gold core–shell nanoparticles are used in PET for dendritic cell labelling and tracking, because it shares high radiosensitivity, desirable labelling efficiency and excellent chemical stability. The novel nanoparticles are also good at monitoring cell biological functions such as proliferation and phenotype marker expression (Lee et al.,2017). Results showed that ^124^I-labelled gold nanostar probes could be used for brain tumors in PET. ^124^I-labelled gold nanostar probes can reach sub-mm intracranial for brain tumour detection (Liu et al.,2019). Radioactive [^64^ *Cu*]*CuS* nanoparticles is also used for PET. Especially when they were conjugated to RGDfK peptides by PEG linkers, they are equipped with a robust ability to target tumor and allowed to be uptaken by tumors. Thus, they can be used to enhance PET for realizing theranostic application (Cui et al.,2018). Very recently, pharmacokinetically-optimized ^64^Cu-labelled polyglucose nanoparticles (Macrin) were developed for quantitative PET imaging of macrophages (Kim et al.,2018).

Besides above gold and copper sulfide nanoparticles, there are many other nanoparticles that can be used in PET, *e.g.*, ^11^C, ^13^N, ^15^O and ^18^F are also radioactive contrast agents for PET. Typically, ^18^F-Macroflor modified polyglucose nanoparticles have a relatively high avidity for macrophages. They are small and excreted renally, thus increasing the PET signal (Keliher et al.,2017).

### Photoacoustic Imaging

**FIGURE 3:**
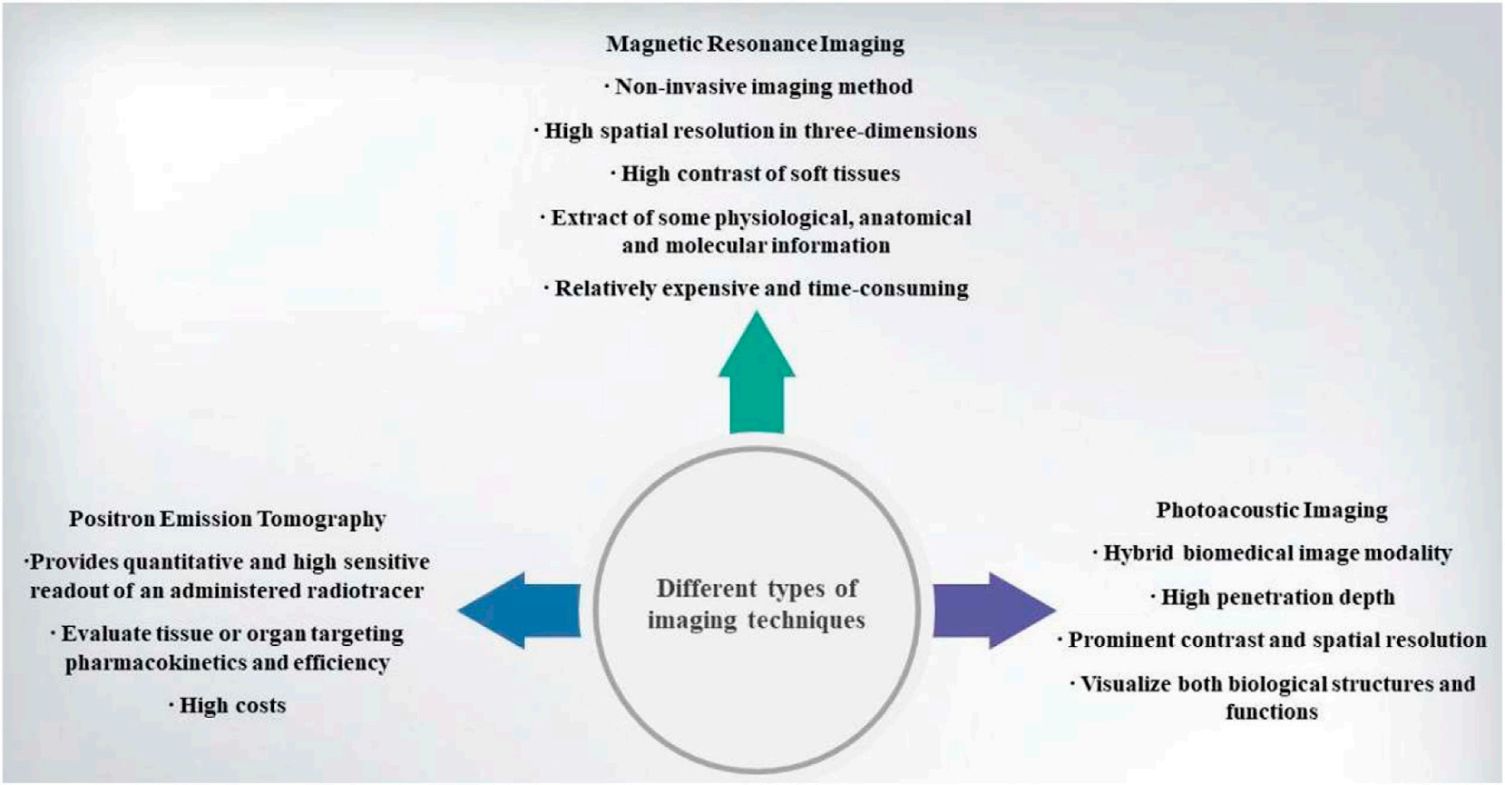
Diagram of the different types of imaging techniques.

Based on photoacoustic effect, photoacoustic imaging is a promising branch of ultrasound or optical modalities. Photoacoustic effect is a part of non-ionizing laser pulse which is absorbed by tissues and then converted into heat. Due to transient thermoelastic expansion, there are wideband ultrasonic waves. So photoacoustic imaging is a hybrid biomedical imaging modality. Ultrasonic images can be made by the generated ultrasonic waves in photoacousic imaging (Zhang et al.,2006). It is an excellent method and outperforms optical imaging in visualizing both biological structures and functions due to its penetration depth, prominent contrast and spatial resolution (Figure 3) (Wang et al.,2003; Yao & Wang, 2000).

Some exogenous agents can improve the photoacoustic imaging. Generally, the most important abilities of PAI contrast agents is to convert the light into heat and produce ultrasound waves. Almost all photothermal materials such as carbon, silver, gold, *etc.*, have this ability. After reconstructing, these materials can distinguish cancer tissues from healthy tissues. Gold-based nanomaterials are promising PAI contrast agents because of their localized surface plasmon resonance effect and can convert light into heat efficiently. The surface of gold is inert, determining the biocompatibility in *vivo* research. Li et al. constructed PEG-HAuNS with both ultrasound and optical properties as contrast agents. This nanomaterial has no acute toxicity in many organs. Its properties in spatial resolution are admirable and good for photoacoustic imaging (Lu et al.,2010). The silver is also known as high surface plasmon resonance. Due to stronger light absorption, silver is theoretically preferable than gold in constructing photoacoustic contrast agent. However, they are more cytotoxic *in vivo*. So, silver-based nanomaterials demand more studies to address their biocompatibility. Carbon-based materials have high resolution and allow imaging of deep areas, holding high potential in clinical translation. Gambhir et al. constructed single-walled carbon nanotubes with a cyclic Arg-Gly-Asp (RGD) peptide as agents in photoacoustic imaging (De la Zerda et al., 2008).

## Cancer Therapy Based on Nanomaterials

### Chemotherapy

Chemotherapy is always used in treating metastatic types of cancers (Wang et al.,2017). Doxorubicin (DOX) and paclitaxel are popular chemotherapeutic drugs which can be used in breast cancer. Chemotherapy regimens such as tamoxifen, trastuzumab, docetaxel and cisplatin are also used in chemotherapy (Siddique & Chow, 2020). Nanoparticle-based carriers conjugating with these chemotherapeutic drugs are often used in targeted delivery. For example, nanoparticles based on polymer, metal, mesoporous silica, protein and carbon, are used in chemotherapy (Liyanage et al.,2019). Different kinds of proteins and peptides are combined with nanoparticles to help improve selectivity of chemotherapeutice drugs.

Gold nanoparticles have distinctive characteristics such as multifunctionalities, high stability, high surface plasmon resonance and large surface area-to-volume ratio. Importantly, Au nanoparticles exhibit additional benefits such as nonimmunogenic nature, nontoxic, high permeability and retention effect. Thus Au nanoparticles carrier enabled penetration and accumulation of chemotherapeutic drugs at tumor areas. Typically, EpCAM-RPAnN and DOX-BLM-PEG-Au NPs are Au nanoparticle-based drug delivery systems that can be applied into chemotherapy (Singh et al.,2018). As well, organic nanoparticles are also good choice for drug delivery in chemotherapy since they can increase tumor accumulation of drugs and prolong their circulation half-time. Briefly, nanoparticles can behave as theranostic platform and deliver many drugs to targeted area (Meng et al.,2017).

### Radiotherapy

Radiotherapy is also primarily used for various nonmetastatic cancers (Wang et al.,2017). Nanovectorized radiotherapy based on nanoparticles for radionuclides delivery can serve as a reservoir to supply radionuclides to cancer cells specifically. This novel method stimulates immune response and cell killing by using peculiar biomaterials (Vanpouille-Box et al.,2011). Many studies have found that nanomaterials can significantly increase the efficiency of radiotherapy.

Nanomaterials have many advantages, such as high toleration, low toxicity and long circulation time. Some of nanomaterials can even act as radioprotectors when exposed to radioactive substances (Nambiar & Yeow, 2012). Colon et al. indicated that cerium oxide (CeO_2_) nanoparticles protected the cells from radiation-induced damages both *in vivo* and *in vitro* (Colon et al.,2009; Heckert et al.,2008). Schweitzer et al. synthesized melanin-covered nanoparticles and found that they could provide a new way to protect bone marrow from ionizing radiation (Schweitzer et al.,2010). Brown et al. found that fullerene compound DF-1 could serve as a radiation protector. As a radiation protector, DF-1 has modest activity *in vivo* and could reduce DNA double strand (Brown et al.,2010).

### Photothermal Therapy

Photothermal therapy (PTT) is a cancer treatment method based on hyperthermia. It can destroy tumor cells and simultaneously avoid to heat normal areas. Nanomaterials such as nanocages, nanoshells, nanostars and nanocages are mostly used as photothermal transducers. Au nanoparticles and near-infrared (NIR) can be used to selectively and precisely heat tumors (Hirschberg & Madsen, 2017). With rich amine and thiol groups, gold nanoparticles can be used as drug products to realize targeted delivery when combining with antibodies. Colloidal gold always localizes plasmon surface resonance, thus absorbing light with specific wavelengths. In this regard, it is helpful in hyperthermic cancer treatment. As the shape or size of particles change, the localized plasmon surface resonance of gold nanoparticles can also be tuned. Accordingly, the properties of photothermal and photoacoustic can be altered (Vines et al.,2019).

PTT suffers from one major problem that is the heterogeneous heat distribution in tumors, which thus leaves some tumor area untreated and causes incomplete ablation. Silica-coated gold nanoshells are proposed to deliver fractionated PTT (Simon et al.,2019). PTT’s main mediators are gold nanoshells due to their high photothermal conversion efficient, excellent biocompatibility, facile gold thiol bioconjugation chemistry and high tumor penetration. The temperature at the tumor is raised aboveb42°C in order to destroy cancer cells. Therefore, a light-absorbing material should improve the energy selectivity for heat transduction. Although gold is mostly used in PTT, magnetic nanoparticles are good alternatives (Siddique & Chow, 2020). Magnetic nanoparticles can combine with other materials or serve as photothermal agents themselves. They can penetrate into tumor area magnetically and their molar absorption coefficient in NIR is low when used alone. Magnetic nanoparticles can also be used in drug delivery, which enables the marriage of PTT and chemotherapy (Estelrich & Busquets, 2018).

### Photodynamic Therapy

Photodynamic therapy (PDT) is a combination of light and photosensitizers. When exposed to light, photosensitizer is able to convert light into reactive oxygen species (ROS) and kill tumor cells through necrosis or apoptosis (Celli et al.,2010). PDT can exert the potent effects to destroy cells and vasculature of the tumor. Because of the light-absorbing photosensitizer’s special and designated localization, the subsequent biological responses can happen in those designated areas (Brown et al.,2004). PDT has potential in clinical cancer treatment with reduced side effects.

**FIGURE 4:**
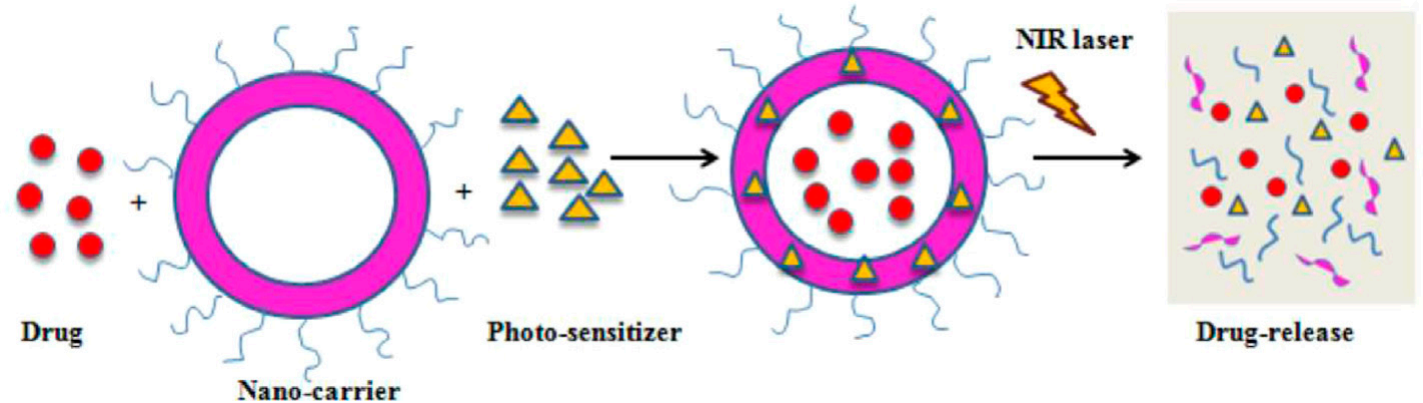
A schematic illustration of PDT-mediated drug release. Reprinted (adapted) with permission from Rai et al. (2021). Medicina (Kaunas). 2021,57 (2),91. Copyright 2021 by the authors.

**FIGURE 5:**
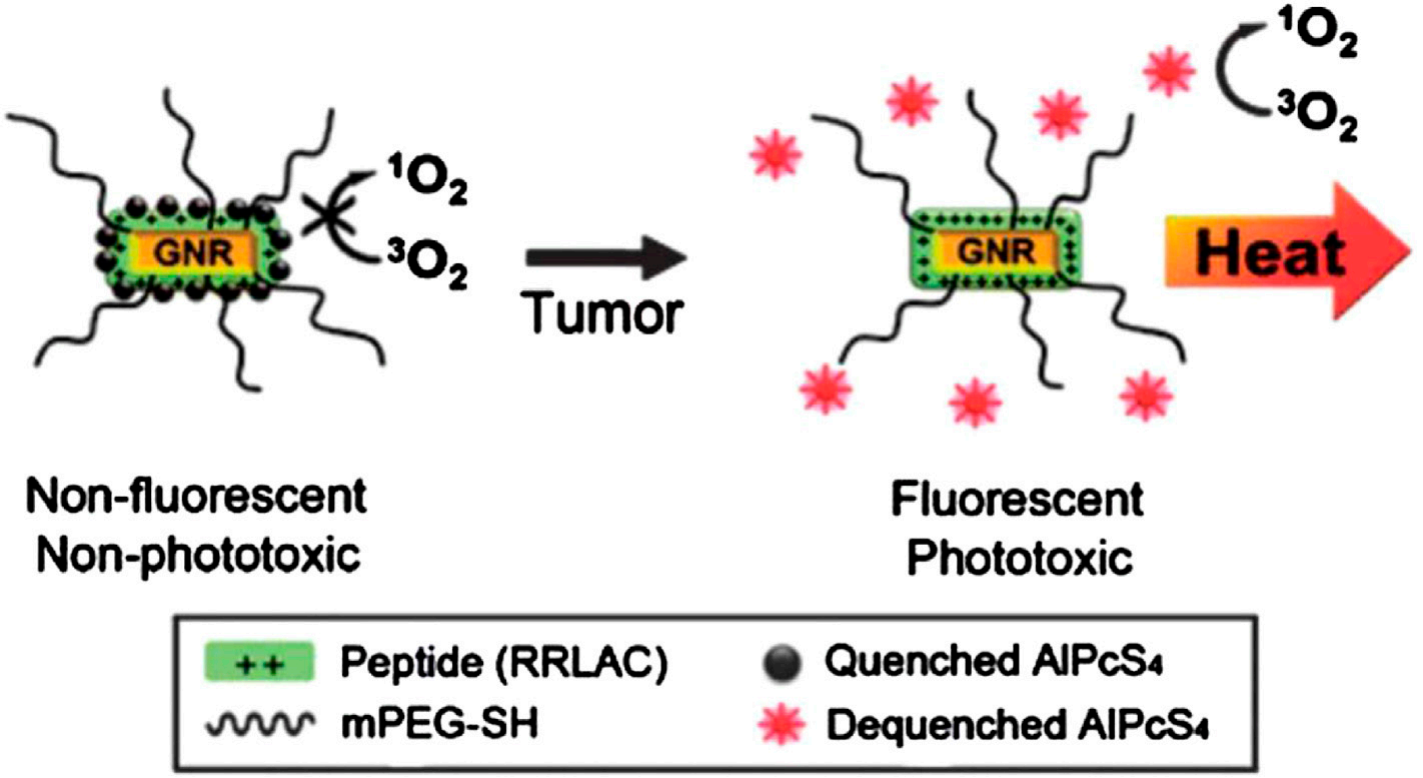
A schematic diagram of the gold nanorods which conjugated with AlPcS4 for simultaneous NIR optical imaging and phototherapy. Reprinted (adapted) with permission from Jang et al. (2011). ACS Nano. 2011,22,5 (2),1,086-94. Copyright 2011 American Chemical Society.

Imaging is added as a direct guidance for localizing photosensitizers. Rai et al. integrated PDT with imaging using the NIR-light absorbed photosensitive molecules (Figure 4)(Rai et al.,2021). So far, a variety of nanomaterials have been developed to behave as carriers to enhance PDT such as semiconductor quantum dots, silica nanoparticles, nanocapsules, gold-based nanomaterials and some metal-based nanomaterials (Gary-Bobo et al.,2011; Son et al.,2011). Gold-based nanomaterials have excellent characterisitics for both PDT and optical imaging. Choi et al. proposed a nanomedicine platform consisting of photosensitizer AlPcS4 and gold nanorods (Jang et al.,2011). Based on this nanoplatform, tumor areas can be discerned by NIR fluorescence imaging, and then dual PDT and PTT was carried out effectively ablate them (Figure 5) (Jang et al.,2011). Wang et al. used photosensitizer Chlorine6 (Ce6) to conjugate with NIR light-excited upconversion nanoparticles (UCNPs) for tumor therapy (Wang et al.,2011).

## Nanomaterials’ Theranostics Application in Multimodality Imaging, Image-Guided Therapy and Combination Therapy

Multimodal imaging that uses nanomaterials can combine their personal advantages and also overcome limitations of each single imaging modality (Rosenkrans et al.,2021). PET-MRI combination perfectly integrates high sensitivity of PET with the excellent spatial resolution and contrast of soft tissue in MRI. SPIONs, Feraheme (FH) and [^89^Zr]Zr were used for PET-MRI as an integral nanoplatform (Gholami et al.,2020), wherein FH shorten *T*_2_ transverse relaxation time for negative contrast enhancement. In addition, ^*68*^Ga-AGulX@NODAGA showed high potential in PET/MRI-guided radiotherapy (Thomas et al.,2017). They not only behave as a dual-modal imaging agent, but also serve as an interstatial radiotherapy agent which can accumulate in the targeted diseased area (Thomas et al.,2017).

Imaging-guided radiotherapy is effective in treating cancer (Chow and Albayedh, 2018; Martelli & Chow, 2020). Combination of gadolinium and bismuth as a theranostic platform can be used for onsite radiosensitization and contrast enhancement. As well, there are other candidates for combination therapy, *e.g.*, mesoporous silica nanoparticles can serve as CT and optical imaging agents. The high density of platinum nanoparticles could significantly enhance CT contrast, and nontoxic Co^2+^ ions were also used as radioactive tracers (Pham et al.,2017).

Combination therapy can cause synergistic actions and work against drug resistance. Several nanoparticles can be used in combined therapy. Liposomes that are established as drug delivery vehicles have various clinical products. Polymeric nanoparticles are also used in combination therapy, and they can be used to deliver site-specific anticancers to tumors. Other nanoparticles such as dendrimers, carbon nanoparticles, metallic nanoparticles and nanodiamonds can be used in combination therapy (Gurunathan et al.,2018).

**FIGURE 6:**
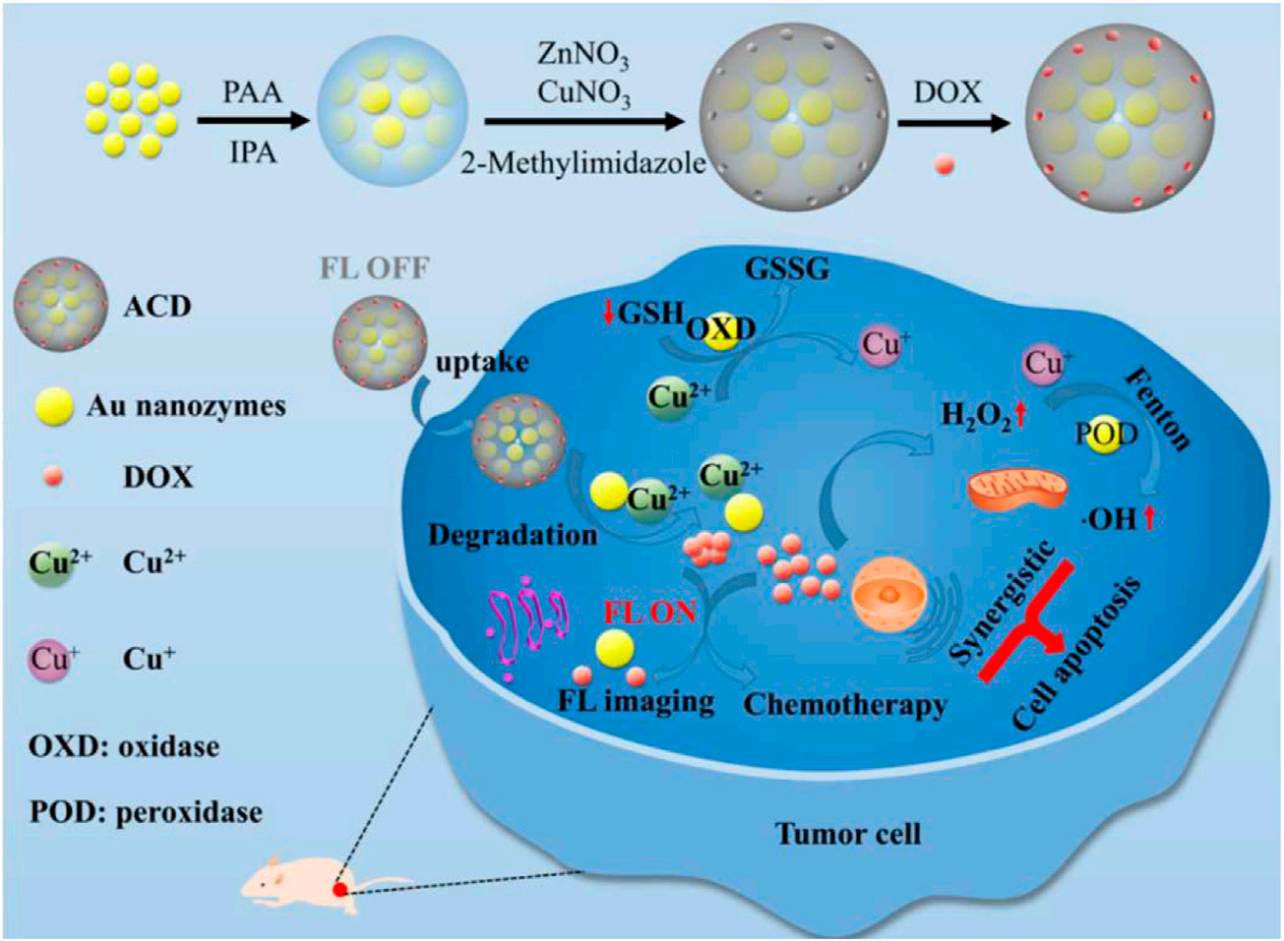
A schematic diagram of fabrication procedure of ACD (ACD, A: Aunanoclusters, C: copper ions, D: DOX) and its use for both fluorescence imaging and enhancing chemo-chemodynamic therapy. Reprinted (adapted) with permission from Zhao et al. (2021). ACS Appl Mater Interfaces. 2021,1,13 (47):55,780-55,789. Copyright 2021 American Chemical Society.

Chemotherapy is a common cancer treatment method. However, it shows off-target effect and cause toxicity in normal issues. Almost 90% of cancer treatment failures is attributed to the chemoresistance. Some tumor cells such as cancer stem cells or progenitor cells have potent radioresistance against many chemotherapeutic agents. Inspiringly, PTT combination with chemotherapy arouses an effect of triggering powerful antitumor immunity. Especially, PTT combination with immunotherapy can further minimize the risk of metastasis or recurrence. As the paradigms, polydopamine-coated spiky Au nanoparticles have high photothermal stability and can be used in PTT-chemotherapy; and polydopamine-coated AI_2_O_3_ nanoparticles can be used for PTT-immunotherapy (Chen et al., 2018). Fluorescence imaging has high resolution and sensitivity. It is real-time imaging capture and is suitable for diagnosing tumors (Zhong, 2009). Chemodynamic therapy is a specific tumor treatment and can make tumor cells sensitive to chemotherapy drugs thus improving the chemo therapy effect (Wang et al.,2018). Recent study indicated that a novel theranostics nanozymes can be used for both fluorescence imaging and enhancing chemo-chemodynamic therapy (Figure 6) (Zhao et al.,2021).

## Conclusion and Perspectives

Nanomaterials are useful tool for cancer diagnosis and therapy. Due to their excellent cellular uptake, targeted drug delivery, high drug loading, prolonged circulation time, high surface to volume ratio, good permeability and retention effect, nanomaterials can enhance the therapeutic effect and reduce the side effects at the same time. In this review, we overviewed different kinds of nanomaterials and explained the latest research on the use of bioresponsive nanomaterials for cancer imaging modalities (magnetic resonance imaging, positron emission tomography and photoacoustic imaging) and imaging-guided therapy (chemotherapy, radiotherapy, photothermal therapy and photodynamic therapy). Nanomaterials are also promising labeling agents for biosensing. Fluorescence-based detection is always used in biosensing due to its diversity, simplicity and sensitivity. Nanomaterials can offer superior optical and acoustic properties, e.g., bright fluorescence and robust acoustic scattering, which can be engineered to realize multiple treatments such as sonodynamic therapy (SDT) (Zhang et al., 2021).

In last few decades, nanomaterials were conjugated with different membranes and biomaterials in order to improve biocompatibility and overcome drug resistance which were observed in cancer treatment commonly. Despite all advantages, it is still a great challenge to use nanomaterials in clinical trials because of their unknown toxicity, cytotoxicity, biocompatibility, *in vivo* targeting efficiency, physicochemical properties, production costs and so on. Novel nanoenzyme is a catalytic nanomaterial with enzymatic properties. It can be used for fluorescence imaging and enhanced chemo-chemodynamic therapy of tumors (Lee et al., 2022). It is still expected that new developments of nanotechnology can happen in nanomaterials in clinics in the near future.
